# Association of Hypertensive Disorders of Pregnancy With Future Cardiovascular Disease

**DOI:** 10.1001/jamanetworkopen.2023.0034

**Published:** 2023-02-17

**Authors:** Bilal Rayes, Maddalena Ardissino, Eric A. W. Slob, Kiran Haresh Kumar Patel, Joanna Girling, Fu Siong Ng

**Affiliations:** 1 National Heart and Lung Institute, Imperial College London, London, United Kingdom; 2 Nuffield Department of Population Health, University of Oxford, Oxford, United Kingdom; 3 Medical Research Council Biostatistics Unit, University of Cambridge, Cambridge, United Kingdom; 4 Department of Applied Economics, Erasmus School of Economics, Erasmus University Rotterdam, Rotterdam, the Netherlands; 5 Erasmus University Rotterdam Institute for Behavior and Biology, Erasmus University Rotterdam, Rotterdam, the Netherlands; 6 Department of Obstetrics and Gynaecology, Chelsea and Westminster National Health Service Trust, London, United Kingdom

## Abstract

**Importance:**

Hypertensive disorders in pregnancy (HDPs) are major causes of maternal and fetal morbidity and are observationally associated with future maternal risk of cardiovascular disease. However, observational results may be subject to residual confounding and bias.

**Objective:**

To investigate the association of HDPs with multiple cardiovascular diseases.

**Design, Setting, and Participants:**

A genome-wide genetic association study using mendelian randomization (MR) was performed from February 16 to March 4, 2022. Primary analysis was conducted using inverse-variance-weighted MR. Mediation analyses were performed using a multivariable MR framework. All studies included patients predominantly of European ancestry. Female-specific summary-level data from FinnGen (sixth release).

**Exposures:**

Uncorrelated (*r*^2^<0.001) single-nucleotide variants (SNVs) were selected as instrumental variants from the FinnGen consortium summary statistics for exposures of any HDP, gestational hypertension, and preeclampsia or eclampsia.

**Main Outcomes and Measures:**

Genetic association estimates for outcomes were extracted from genome-wide association studies of 122 733 cases for coronary artery disease, 34 217 cases for ischemic stroke, 47 309 cases for heart failure, and 60 620 cases for atrial fibrillation.

**Results:**

Genetically predicted HDPs were associated with a higher risk of coronary artery disease (odds ratio [OR], 1.24; 95% CI, 1.08-1.43; *P* = .002); this association was evident for both gestational hypertension (OR, 1.08; 95% CI, 1.00-1.17; *P* = .04) and preeclampsia/eclampsia (OR, 1.06; 95% CI, 1.01-1.12; *P* = .03). Genetically predicted HDPs were also associated with a higher risk of ischemic stroke (OR, 1.27; 95% CI, 1.12-1.44; *P* = 2.87 × 10^−4^). Mediation analysis revealed a partial attenuation of the effect of HDPs on coronary artery disease after adjustment for systolic blood pressure (total effect OR, 1.24; direct effect OR, 1.10; 95% CI, 1.02-1.08; *P* = .02) and type 2 diabetes (total effect OR, 1.24; direct effect OR, 1.16; 95% CI, 1.04-1.29; *P* = .008). No associations were noted between genetically predicted HDPs and heart failure (OR, 0.97; 95% CI, 0.76-1.23; *P* = .79) or atrial fibrillation (OR, 1.11; 95% CI, 0.65-1.88; *P* = .71).

**Conclusions and Relevance:**

The findings of this study provide genetic evidence supporting an association between HDPs and higher risk of coronary artery disease and stroke, which is only partially mediated by cardiometabolic factors. This supports classification of HDPs as risk factors for cardiovascular disease.

## Key Points

**Question:**

Is there evidence for an association between hypertensive disorders of pregnancy (HDPs) and long-term risk of cardiovascular disease?

**Findings:**

In this large genome-wide genetic association study using mendelian randomization, HDPs were associated with higher risk of coronary artery disease and ischemic stroke but not heart failure or atrial fibrillation. Mediation analysis revealed a partial attenuation of the association between HDPs and coronary artery disease after adjustment for systolic blood pressure and type 2 diabetes.

**Meaning:**

These results support the consideration of HDPs as potential risk factors for cardiovascular disease.

## Teaser

This genetic association study uses mendelian randomization to examine factors associated with long-term outcomes of hypertensive disorders in pregnancy.

## Introduction

Hypertensive disorders in pregnancy (HDPs) affect nearly 10% of pregnancies and account for approximately 14% of maternal deaths worldwide.^1,2^ Globally, HDPs are the second leading cause of maternal mortality and a major cause of neonatal morbidity.^1^

The term *hypertensive disorders* encompasses 3 diagnoses: gestational hypertension, preeclampsia or eclampsia, and either of these pathologic conditions superimposed on chronic hypertension.^3^ Beyond their short-term impact on maternal and fetal outcomes of pregnancy, HDPs also have implications for long-term maternal health. Observational evidence suggests that women who experience HDPs have a 2-fold higher long-term risk of future cardiovascular events compared with women who have normotensive pregnancy.^4,5^ In addition, recent data from the Nurses’ Health Study II cohort revealed that women with HDPs had a significantly higher rate of atherosclerosis in the years following pregnancy.^6^

However, causal inference cannot be drawn from such observational associations due to the potential residual impact of confounding. This is particularly important for HDPs, as there are many potential common causes of HDPs and cardiovascular disease. These include clinical factors, such as diabetes^7^ and obesity,^7,8^ and socioeconomic and behavioral factors that are extremely difficult to quantify and account for in an observational setting.^9,10^

Mendelian randomization (MR) is a method that uses genetic risk of disease as a proxy for the disease itself to use in an instrumental variant analysis framework.^11^ Like randomization to treatment in a clinical trial, genetic variants are randomly assigned at the time of gamete formation and conception, independent of external influences. This leads to effective randomization to either high or low genetic risk of a disease, mitigating the potential for confounding and reverse causation similar to a randomized clinical trial. Under a set of assumptions, MR estimates can be interpreted as the estimated effect of the exposure on the outcome.

The aim of this study was to use MR to investigate the association of HDPs (when considered as a whole and as the individual diagnoses of gestational hypertension and preeclampsia or eclampsia) with the risk of coronary artery disease (CAD), ischemic stroke, heart failure, and atrial fibrillation. Furthermore, we explored the role of traditional cardiovascular risk factors as potential mediators of this association.

## Methods

### Study Data

All data used in this genome-wide genetic association study using mendelian randomization are deidentified publicly available. All cited data sources obtained participant informed consent and relevant ethical approval. The study was conducted from February 16 to March 4, 2022. Details of the studies used as data sources are outlined in eTable 1 in Supplement 1. This study is reported following recommendations by the Strengthening the Reporting of Observational Studies in Epidemiology Using Mendelian Randomization (STROBE-MR) reporting guideline.^12^

### Instrumental Variants for Exposures

This study considered 3 exposures: HDPs as a whole and its 2 subtypes of gestational hypertension and preeclampsia/eclampsia. Genetic association estimates were extracted from the FinnGen consortium’s sixth data release of genome-wide association study (GWAS) summary data, released on January 24, 2022.^13^ This included 10 736 cases and 136 325 controls for any HDPs, 5240 cases and 136 325 controls for gestational hypertension, and 4743 cases and 136 325 controls for preeclampsia/eclampsia, with a mean age at first event between 29.06 and 30.08 years.

Instrumental variants were selected if they were associated with each exposure at a genome-wide significance threshold of *P* < 5 × 10^−8^. No single-nucleotide variants (SNVs) were available at this threshold for preeclampsia/eclampsia, so the threshold was increased by a factor of 10 until at least 1 variant was available, resulting in a final *P* value threshold for preeclampsia/eclampsia of *P* < 5 × 10^−6^. A list of instrumental variants used in the analysis for the exposures can be found in eTable 3, eTable 4, and eTable 5 in Supplement 1.

### Genetic Associations for Outcomes

Genetic association estimates for the outcome of CAD were obtained from the van der Harst and Verweij^14^ study of 122 733 cases and 424 528 controls. Genetic association estimates for ischemic stroke (IS) were obtained from the Malik et al^15^ study of 34 217 cases and 406 111 controls. Genetic association estimates were obtained from the Shah et al^16^ investigation of 47 309 cases and 930 014 controls for heart failure (HF) and the Nielsen et al^17^ study of 60 620 cases and 970 216 controls for atrial fibrillation (AF). All studies were carried out in European ancestry populations, and genetic association estimates were adjusted for participant sex. Further details on population characteristics are available in the original publications and eTable 1 in Supplement 1.

### Harmonization and Clumping

**Figure 1.**
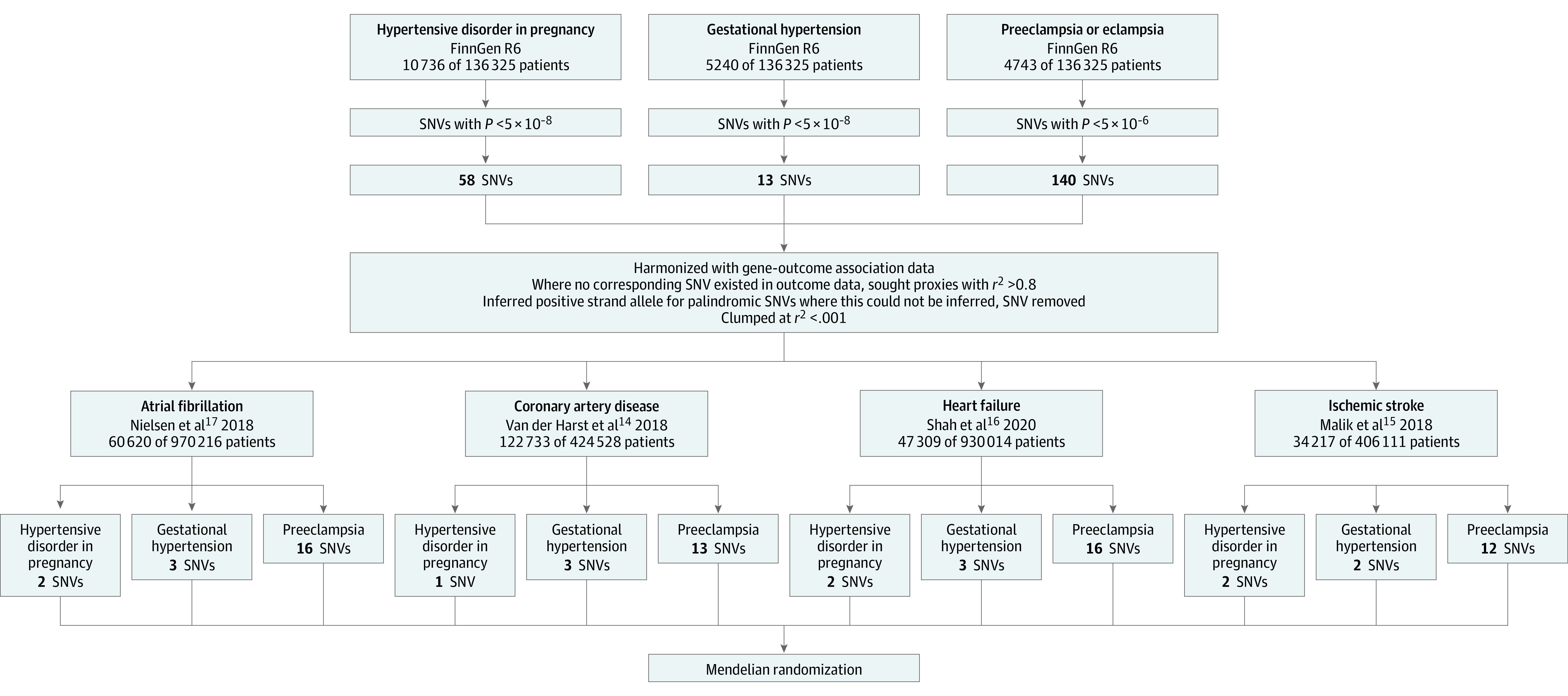
Study Data, Instrumental Variant Selection, and Analysis Flowchart Genome-wide association study summary data were acquired from the FinnGen consortium on the 3 primary exposures. Mendelian randomization analysis was conducted for the outcomes of coronary artery disease, heart failure, atrial fibrillation, and acute ischemic stroke. R6 indicates release 6; SNV, single-nucleotide variant.

Estimates of the effect of instrumental variant SNVs for each exposure were harmonized with effect estimates of the SNVs for outcomes, separately for each exposure-outcome pair. If there were no matching SNVs for an instrumental variant in the outcome summary statistics, proxies were sought that were in linkage disequilibrium with an *r*^2^ value greater than 0.8. Clumping was performed after harmonization of data using the TwoSampleMR package in R software at *r*^2 ^less than 0.001. The flowchart to show data sources, instrumental variant selection, and statistical analysis is shown in Figure 1.

### Statistical Analysis

The primary method used for analysis was inverse-variance-weighted MR with multiplicative random effects^18^ in all instances when 4 or more SNVs were available. If 2 or 3 SNVs were available, inverse-variance-weighted MR with fixed random effects was used. If only 1 instrumental SNV was available, the Wald ratio was used. Heterogeneity in inverse-variance-weighted analyses was estimated using the Cochran *Q* statistic.

Single SNV analyses were performed using the Wald ratio method, and leave-1-out analyses were also conducted by performing multiple analyses after sequentially removing 1 SNV from the instrumental variant set. This could only be carried out when 3 or more instrumental variants were present.

Additional sensitivity analyses were performed to assess and address the key MR assumptions regarding instrumental variants, described in further detail in the eMethods in Supplement 1. The first assumption was explored by quantifying the strength of instruments using *R*^2^ and *F* statistics.^19^ Weighted median MR,^20^ MR-Egger regression,^21^ and MR-PRESSO^22^ were performed to identify and account for potential horizontal pleiotropy.^23^ All statistical analysis was conducted in R, version 4.1.2 (R Foundation for Statistical Computing) using TwoSampleMR^24^ and Mendelianrandomization packages.^25^

For any exposure-outcome associations significant in primary analysis, mediation analysis was performed using multivariable MR.^26^ The putative mediators considered included body mass index^27^ (434 749 individuals of European ancestry), systolic blood pressure^28^ (757 601 individuals of European ancestry), and type 2 diabetes ^29^ (80 154 individuals and 853 816 controls of European ancestry). These were chosen on the basis of a large, recent observational study that reported different trajectories in these factors among women with HDPs.^6^

A step-wise approach was used. First, statistically significant (*P* < .05) exposure-outcome associations were identified. Second, exposure-mediator associations were tested using univariable inverse-variance-weighted MR. Only putative mediators that were associated with the exposure (downstream, at a less conservative statistical significance level of *P* < .10) were carried forward. We opted for a less stringent threshold here since, in some conditions, mediation can be true despite not finding an association between the exposure and mediator.^30^ In addition, the direct effect of the exposure on the outcome, conditional on the mediator, was calculated using multivariable MR. Since the outcomes in our MR investigation are common and since binary variables and genetic association estimates are in log(OR) scale, which is noncollapsible in the setting of a common outcome, the indirect effect was not reported. Instead, by qualitatively comparing the direct and total effects, any significant attenuation of the odds ratios (ORs) after conditioning on a mediator was taken to indicate the presence of a mediating pathway.^31^ The conditional *F* statistic was calculated to assess the strength of instruments in multivariable MR analysis.^32^

Results are presented as ORs with 95% (CIs). Statistical significance was considered at a 2-sided α level threshold of *P* < .05. Post hoc power calculations for univariable primary analyses were performed using the online mRnd calculator to estimate the minimum effect size that we had at least 80% power to detect.^33^

## Results

### Coronary Artery Disease

**Figure 2.**
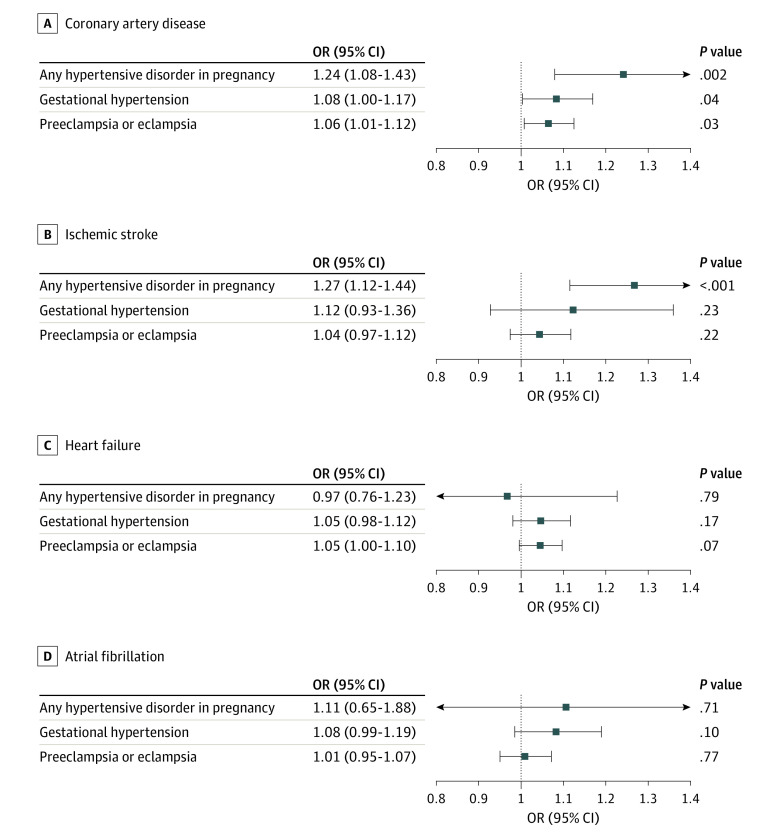
Mendelian Randomization Analysis of Hypertensive Disorders of Pregnancy and Cardiovascular Outcomes Mendelian randomization estimates for the association of any hypertensive disorder in pregnancy, gestational hypertension, and preeclampsia/eclampsia with the outcomes of (A) coronary artery disease, (B) acute ischemic stroke, (C) heart failure, and (D) atrial fibrillation. OR indicates odds ratio.

Genetically predicted HDPs (OR, 1.24; 95% CI, 1.08-1.43; *P* = .002), genetically predicted gestational hypertension (OR, 1.08; 95% CI, 1.00-1.17; *P* = .04), and genetically predicted preeclampsia/eclampsia (OR, 1.06; 95% CI, 1.01-1.12; *P* = .03) were associated with a higher risk of CAD, as illustrated in Figure 2 and Table 1. Sensitivity analyses revealed no evidence of directional pleiotropy for either genetically predicted gestational hypertension (MR-Egger intercept *P* = .33) or genetically predicted preeclampsia/eclampsia (MR-Egger intercept *P* = .71), although some heterogeneity was noted (HDPs *Q* statistic = 13.6; *P* = .001; gestational hypertension *Q* statistic = 3.8; *P* = .15; and preeclampsia/eclampsia *Q* statistic = 32.3; *P* = .004). MR-PRESSO did not identify a significant impact of outliers on results but could only be done for preeclampsia/eclampsia as the analysis requires more than 3 SNVs. Sensitivity analyses could not be carried out for genetically predicted HDPs, as only 1 SNV was available for analysis. The results of single SNV analysis are reported in eFigure 1 in Supplement 1, and leave-1-out analyses are reported in eTable 6 in Supplement 1.

**Table 1.** MR Estimates for the Effect of Any Hypertension in Pregnancy, Gestational Hypertension, and Preeclampsia/Eclampsia on CAD, IS, HF, and AF

Exposure and outcome	MR method	No. of SNVs	OR (95% CI)	*P* value
Any hypertensive disorder in pregnancy				
CAD	Wald ratio	1	1.24 (1.08-1.43)	.002
IS	IVW	2	1.27 (1.12-1.44)	2.86 × 10^−4^
HF	IVW	2	0.97 (0.76-1.23)	.79
AF	IVW	2	1.11 (0.65-1.88)	.71
Gestational hypertension				
CAD	IVW	3	1.08 (1.00-1.17)	.04
	Weighted median	3	1.10 (1.02-1.18)	.01
	MR-Egger^a^	3	0.98 (0.87-1.11)	.83
IS	IVW	2	1.12 (0.93-1.36)	.23
HF	IVW	3	1.05 (0.98-1.12)	.17
	Weighted median	3	1.06 (0.98-1.15)	.16
	MR-Egger^b^	3	0.97 (0.84-1.12)	.78
AF	IVW	3	1.08 (0.99-1.19)	.10
	Weighted median	3	1.07 (1.00-1.15)	.08
	MR-Egger^c^	3	0.95 (0.84-1.07)	.54
Preeclampsia/eclampsia				
CAD	IVW	13	1.06 (1.01-1.12)	.03
	Weighted median	13	1.03 (0.97-1.09)	.38
	MR-Egger^d^	13	1.05 (0.94-1.17)	.44
	MR PRESSO		1.07 (1.01-1.13)	.046
IS	IVW	12	1.04 (0.97-1.12)	.22
	Weighted median	12	1.01 (0.94-1.08)	.79
	MR-Egger^e^	12	1.00 (0.82-1.22)	.99
	MR PRESSO		1.02 (0.96-1.07)	.57
HF	IVW	16	1.05 (1.00-1.10)	.07
	Weighted median	16	1.04 (0.98-1.11)	.17
	MR-Egger^f^	16	1.06 (0.94-1.18)	.37
	MR PRESSO		1.04 (1.00-1.10)	.09
AF	IVW	16	1.01 (0.95-1.07)	.77
	Weighted median	16	1.01 (0.96-1.07)	.63
	MR-Egger^g^	16	1.12 (1.01-1.24)	.05
	MR PRESSO		1.00 (0.96-1.04)	.91

Abbreviations: AF, atrial fibrillation; CAD, coronary artery disease; HF, heart failure; IS, ischemic stroke; IVW, inverse-variance-weighted with mixed random effects; MR, mendelian randomization; OR, odds ratio; SNV, single-nucleotide variant.

^a^ The MR-Egger intercept (SE) was 0.020 (0.011); *P* = .33.

^b^ The MR-Egger intercept (SE) was 0.015 (0.013); *P* = .47.

^c^ The MR-Egger intercept (SE) was 0.028 (0.011); *P* = .25.

^d^ The MR-Egger intercept (SE) was 0.004 (0.010); *P* = .71.

^e^ The MR-Egger intercept (SE) was 0.007 (0.016); *P* = .65.

^f^ The MR-Egger intercept (SE) was −0.002 (0.009); *P* = .85.

^g^ The MR-Egger intercept (SE) was −0.022 (0.009); *P* = .04.

### Ischemic Stroke

As shown in Figure 2 and Table 1, genetically predicted HDPs were associated with a higher risk of IS (OR, 1.27; 95% CI, 1.12-1.44; *P* = 2.86 × 10^−4^). However, neither subgroups of genetically predicted gestational hypertension (OR, 1.12; 95% CI, 0.93-1.36; *P* = .23) nor genetically predicted preeclampsia/eclampsia (OR, 1.04; 95% CI, 0.97-1.12; *P* = .22) showed any association with IS.

Sensitivity analyses revealed no evidence of directional pleiotropy for genetically predicted preeclampsia/eclampsia (MR-Egger *P* = .65). Sensitivity analyses could not be carried out for HDPs and gestational hypertension because there were insufficient SNVs available for analysis. The full results are given in Table 1. Some heterogeneity was noted for gestational hypertension (*Q* statistic = 5.4; *P* = .02) and preeclampsia/eclampsia (*Q* statistic = 23.8; *P* = .01) but not for HDPs (*Q* statistic = 2.0; *P* = .37). The results of single SNV analysis are reported in eFigure 2 in Supplement 1, and leave-1-out analyses are noted in eTable 6 in Supplement 1.

### Heart Failure

Genetically predicted HDPs (OR, 0.97; 95% CI, 0.76-1.23; *P* = .79), genetically predicted gestational hypertension (OR, 1.05; 95% CI, 0.98-1.12; *P* = .17), and genetically predicted preeclampsia/eclampsia (OR, 1.05; 95% CI, 1.00-1.10; *P* = .07) were not associated with HF, as displayed in Figure 2 and Table 1. Some heterogeneity was noted for HDPs (*Q* statistic = 11.8; *P* = .008) but not for gestational hypertension (*Q* statistic = 1.5; *P* = .48) and preeclampsia/eclampsia (*Q* statistic = 23.5; *P* = .10). The results of single SNV analysis are reported in eFigure 3 in Supplement 1, and leave-1-out analyses are given in eTable 6 in Supplement 1.

### Atrial Fibrillation

As shown in Figure 2 and Table 1, genetically predicted HDPs (OR, 1.11; 95% CI, 0.65-1.88; *P* = .71), genetically predicted gestational hypertension (OR, 1.08; 95% CI, 0.99-1.19; *P* = .10), and genetically predicted preeclampsia/eclampsia (OR, 1.01; 95% CI, 0.95-1.07; *P* = .77) were not associated with AF. Significant heterogeneity was noted for HDPs (*Q* statistic = 33.0; *P* = 6.75 × 10^−8^) and preeclampsia/eclampsia (*Q* statistic = 50.0; *P* = 1.23 × 10^−5^) but not for gestational hypertension (*Q* statistic = 5.8; *P* = .06). The results of single SNV analysis are reported in eFigure 4 in Supplement 1, and the results of leave-1-out analyses are reported in eTable 6 in Supplement 1.

### Statistical Power

For each of the outcomes, the relative risk increase that the analyses had at least 80% power to detect is displayed in Table 2. For the exposure of HDPs, there was at least 80% power to detect a relative risk increase of 8.8% for CAD, 15.2% for IS, 11.1% for HF, and 11.3% for AF. For the exposure of gestational hypertension, there was at least 80% power to detect a relative risk increase of 6.9% for CAD, 14.2% for IS, 9.8% for HF, and 8.7% for AF. For the exposure of preeclampsia/eclampsia, there was at least 80% power to detect a relative risk increase of 3.5% for CAD, 6.8% for IS, 4.7% for HF, and 4.3% for AF.

**Table 2.** Post Hoc Power Calculations for Instrumental Variants

Exposure and outcome	%		IV *F* statistic	RRI with 80% power, %^a^
	*r*^2^ for exposure	*I*^2^ for exposure		
**Any hypertensive disorder in pregnancy**				
CAD	1.09	0.97	118	8.8
IS	1.09	0.97	118	15.2
HF	1.43	0.97	156	11.1
AF	1.09	0.97	118	11.3
**Gestational hypertension**				
CAD	1.82	0.50	97.1	6.9
IS	1.26	0.50	66.6	14.2
HF	1.82	0.50	97.1	9.8
AF	1.82	0.50	97.1	8.7
**Preeclampsia/eclampsia**				
CAD	7.01	0.95	357	3.5
IS	5.57	0.95	279	6.8
HF	7.99	0.95	410	4.7
AF	7.48	0.95	382	4.3

Abbreviations: AF, atrial fibrillation; CAD, coronary artery disease; HF, heart failure; IS, ischemic stroke; IV instrumental variant; RRI, relative risk increase.

^a^ The lowest RRI that the analysis had at least 80% power to detect is reported based on the variance in the exposure phenotype explained by the single-nucleotide variants (*R*^2^), the sample size of the outcome study, and a 2-sided α value of .05.

### Mediation Analysis

The results of the univariable analyses used to select potential mediators are reported in eTable 2 in Supplement 1. There were insufficient instruments to assess the exposure-mediator associations for preeclampsia/eclampsia and CAD. Mediation analysis could not be done for the association between HDPs and IS due to insufficient genetic variants.

Mediation analysis for the association between HDPs and CAD revealed a partial attenuation of effect estimates after adjustment for systolic blood pressure (total effect OR, 1.24; 95% CI, 1.08-1.43; direct effect OR, 1.10; 95% CI, 1.02-1.08; *P* = .02) and type 2 diabetes (total effect OR, 1.24; direct effect OR, 1.16; 95% CI, 1.04-1.29; *P* = .008). Residual significant associations of HDPs with CAD were evident in both cases, as noted in Table 3. This suggests that systolic blood pressure and type 2 diabetes partially mediate the association between HDPs and CAD. Conditional *F* statistics for HDPs on CAD were 3.08 after accounting for systolic blood pressure and 16.7 after accounting for type 2 diabetes. This suggests that the instruments for the mediation analysis for systolic blood pressure were relatively weak, and therefore the mediating effect of systolic blood pressure might have some bias due to weak instruments.

**Table 3.** Multivariable Mendelian Randomization for the Association of Any Hypertensive Disorder in Pregancy With Coronary Artery Disease^a^

Mediator	OR (95% CI)	*P* value
None	1.24 (1.08-1.43)	.002
Systolic blood pressure	1.10 (1.02-1.18)	.02
Type 2 diabetes	1.16 (1.04-1.29)	.008

Abbreviation: OR, odds ratio.

^a^ Evidence of attenuation of the effect estimate toward the null is taken to indicate potential mediation by the factor included in the multivariable analysis.

## Discussion

By using genetic data, we explored the relevance of HDPs on risk of 4 major cardiovascular diseases. Genetically predicted HDPs were associated with higher risk of both CAD and IS. A partial attenuation of effect estimates for the association of HDPs and CAD was seen after adjustment for systolic blood pressure and type 2 diabetes. Furthermore, both HDP subtypes of gestational hypertension and preeclampsia/eclampsia displayed consistent associations with higher risk of CAD. No associations were noted between HDPs and the outcomes of HF or AF. The results of this study add to current literature by providing evidence supporting an association between HDPs and higher risk of atherosclerotic cardiovascular disease.

### Main Findings in Context

There has been growing interest in evaluating the role of HDPs in future maternal cardiovascular risk. Recently, Stuart et al^6^ demonstrated a risk of cardiovascular disease in women with HDPs compared with normotensive controls. In addition, higher rates of subclinical coronary atherosclerosis in women with a history of HDPs have been reported.^34^ However, it is difficult to confidently draw causal inference from observational data due to the potential residual impact of imperfectly or incompletely measured confounding and bias. Our study adds to existing literature through the use of mendelian randomization. Due to the limited impact of confounding and bias in MR, the associations reported support a potentially causal pathway. Our results support the growing acceptance of HDPs as sex-specific risk factors for cardiovascular disease.

The associations with CAD reported in this study appear to be more evident in the global exposure of HDPs and trend toward lower strength for gestational hypertension and even lower strength for preeclampsia and eclampsia, although the findings are still statistically significant. There are a number of potential explanations for this. First, the exposure of HDPs (but neither of the others) included preexistent hypertension; thus, there might be an underlying biological mechanism by which the association is at least in part affected by this subgroup of patients. However, it is difficult to interpret this trend in detail; the methods used across different analyses as well as their underlying power vary significantly across exposures, and therefore differences in apparent strengths of association might be expected.

We did not identify an association between HDPs and HF or AF. In contrast, some observational studies have identified that HDPs, particularly preeclampsia, are associated with higher rates of HF in later life^35,36^ as well as peripartum cardiomyopathy.^37^ The null results may be due to multiple reasons. First, the GWAS data^16^ included all types of HF, and the resulting heterogeneity in the outcome may have reduced the power of our study.^38^ Second, the true effect of HDPs on HF might simply be smaller than what we had the power to detect: we only had 80% or more power to identify an association if there is a true underlying RRI of 11% for HDPs, 10% for gestational hypertension, and 5% for preeclampsia/eclampsia on HF. In addition, the discrepancy might arise from residual confounding in the observational setting. This is particularly true for preeclampsia, which is known to have extensive sociobehavioral and multifactorial influences.^39,40^

### Mediation Analysis

Beyond demonstrating the association of HDPs with cardiovascular disease, it is important to consider underlying biological mechanisms with the aim of identifying actionable treatment targets. We performed mediation analysis using multivariable MR to explore the role of potential mediators of the association between HDPs and CAD. The results revealed only partial mediation by systolic blood pressure and type 2 diabetes. This has important implications: first, it identifies substantial actionable targets for primary prevention, and second, it identifies the presence of a key residual mechanism that is of an unclear source, representing an important target for future research.

The effect of HDPs on CAD was attenuated after adjustment for type 2 diabetes and systolic blood pressure. Timely and effective treatment of these factors is therefore a key strategy to attenuate the higher risk of CAD conferred by HDPs and should be a fundamental clinical priority for patients with a history of HDP. Currently, international guidelines from the American College of Obstetrics and Gynecology,^41^ European Society of Cardiology,^42^ and American Heart Association and American College of Cardiology^43^ highlight the importance of timely screening for cardiovascular risk factors and disease post partum in women with HDP in pregnancy. However, there is little past research evaluating the impact of active primary preventive strategy (eg, continuation of antihypertensive therapy). In addition to this, the implementation of recommendations remains low, with a recent study estimating that only 58% of women with HDP had a follow-up visit with a continuity clinician post partum.^44^ The results of our study may encourage further elucidation of optimal prevention strategies and improved implementation of care pathways in this high-risk cohort.

In this study, the association between HDPs and CAD was only partially mediated by traditional cardiovascular disease risk factors. This suggests that HDPs might increase the risk of CAD through additional mechanisms. There are multiple potential candidate mechanisms that might underpin this residual effect. Preeclamptic placentas have been shown to have an imbalance of reactive oxygen species and circulating angiogenic factors that are associated with endothelial dysfunction.^45^ The resultant endothelial dysfunction might lead to damage that persists after pregnancy and creates a substrate for atherosclerotic plaque formation. Alternatively, the endothelial dysfunction itself might persist subclinically after pregnancy and confer a higher risk of atherosclerosis through ongoing endothelial damage. This is corroborated by the well-known association between HDPs and risk of subsequent hypertension.^46^ Further research in this field is required, as elucidation of mechanisms is a key step to help targeted clinical management and drug development.

### Limitations

Our MR study addresses multiple important limitations of conventional observational studies. Nevertheless, we acknowledge some limitations. First, the GWAS summary data used in this study were from European populations. Second, the analyses only had power to detect differences of a certain size, which we estimated and reported. In addition, although sex-specific GWAS data were used for the exposure, the outcome data were extracted from GWAS summary statistics on both men and women, which was nevertheless sex-adjusted. Also, MR-Egger testing is known to be of limited scope in the setting of few instrumental variants, as was the case for HDPs and gestational hypertension. For this reason, we used multiple sensitivity analyses methods to corroborate the presence or absence of pleiotropy. In the mediation analysis we could not verify that the occurrence of the mediator followed the exposure. Nonetheless, even if these cardiometabolic factors exert their effects on cardiovascular outcomes upstream of HDPs, the fact that the associations remained after adjustment demonstrates that they do not globally account for the association. Finally, it has been shown that for genetic variants whose association with the exposure changes with time, such as systolic blood pressure and body mass index, the MR estimate may not provide a holistic estimate of the lifetime effect.^47^ However, since systolic blood pressure and body mass index were not treated as primary exposures in the study, their impact on the results is minimized.

## Conclusions

We explored the association between HDPs and maternal risk for major cardiovascular diseases. Hypertensive disorders in pregnancy were associated with a higher risk of atherosclerotic cardiovascular diseases, specifically CAD and IS. Cardiometabolic factors mediated part of the association between HDPs and CAD, highlighting key monitoring and treatment targets, but also highlighting a residual effect of a currently unclear source. Broadly, these results support emerging recommendations to consider HDPs as important sex-specific risk factors for atherosclerotic cardiovascular disease.

Hypertensive disorders of pregnancy are associated with CAD and IS. Given that some of this risk is mediated by blood pressure and diabetes, these modifiable risk factors are important targets for primary prevention in women with past HDPs.

Future studies should focus on evaluating the mechanism underlying the direct association between HDPs and CAD. Efforts should also focus on increasing genetic studies of obstetric complications in pregnancy to enable comprehensive assessment of the importance of obstetric morbidity on long-term maternal outcomes.

## Notes

Supplement 1. **eMethods.** Detailed Methods **eTable 1.** Information on the Studies and Consortia From Which Genetic Association Data Were Obtained **eTable 2.** Assessment of Exposure-Mediator Associations **eTable 3.** List of Instrumental Variables Used in the Analyses for the Exposure of Hypertensive Disorders of Pregnancy, and Corresponding Gene-Outcome Association Estimates for All SNPs Available in Outcome GWAS or Proxies Discovered Using a Linkage Disequilibrium Threshold of 0.8 **eTable 4.** List of Instrumental Variables Used in the Analyses for the Exposure of Gestational Hypertension, and Corresponding Gene-Outcome Association Estimates for All SNPs Available in Outcome GWAS or Proxies Discovered Using a Linkage Disequilibrium Threshold of 0.8 **eTable 5.** List of Instrumental Variables Used in the Analyses for the Exposure of Pre-eclampsia or Eclampsia, and Corresponding Gene-Outcome Association Estimates for All SNPs Available in Outcome GWAS or Proxies Discovered Using a Linkage Disequilibrium Threshold of 0.8 **eTable 6.** Results of Leave-1-Out Analyses for All Exposure-Outcome Combinations Where the Number of Instruments Is >2 **eFigure 1.** Forest Plots Showing the Single SNP Analysis for the Exposures of Gestational Hypertension and Pre-eclampsia or Eclampsia and the Outcome of Coronary Artery Disease **eFigure 2.** Forest Plots Showing the Single SNP Analysis for the Exposures of Hypertensive Disorders in Pregnancy, Gestational Hypertension and Pre-eclampsia or Eclampsia and the Outcome of Ischemic Stroke **eFigure 3.** Forest Plots Showing the Single SNP Analysis for the Exposures of Hypertensive Disorders in Pregnancy, Gestational Hypertension and Pre-eclampsia or Eclampsia and the Outcome of Heart Failure **eFigure 4.** Forest Plots Showing the Single SNP Analysis for the Exposures of Hypertensive Disorders in Pregnancy, Gestational Hypertension and Pre-eclampsia or Eclampsia and the Outcome of Atrial Fibrillation

Supplement 2.
Data Sharing Statement
