# Supplementary material for: Association of Hypertensive Disorders of Pregnancy With Future Cardiovascular Disease
Source: JAMA Netw Open. 2023 Feb 17;6(2):e230034. doi: 10.1001/jamanetworkopen.2023.0034 (PMC9938428; doi:10.1001/jamanetworkopen.2023.0034)
Supplement: Supplement 1. — eMethods. Detailed Methods eTable 1. Information on the Studies and Consortia From Which Genetic Association Data Were Obtained eTable 2. Assessment of Exposure-Mediator Associations eTable 3. List of Instrumental Variables Used in the Analyses for the Exposure of Hypertensive Disorders of Pregnancy, and Corresponding Gene-Outcome Association Estimates for All SNPs Available in Outcome GWAS or Proxies Discovered Using a Linkage Disequilibrium Threshold of 0.8 eTable 4. List of Instrumental Variables Used in the Analyses for the Exposure of Gestational Hypertension, and Corresponding Gene-Outcome Association Estimates for All SNPs Available in Outcome GWAS or Proxies Discovered Using a Linkage Disequilibrium Threshold of 0.8 eTable 5. List of Instrumental Variables Used in the Analyses for the Exposure of Pre-eclampsia or Eclampsia, and Corresponding Gene-Outcome Association Estimates for All SNPs Available in Outcome GWAS or Proxies Discovered Using a Linkage Disequilibrium Threshold of 0.8 eTable 6. Results of Leave-1-Out Analyses for All Exposure-Outcome Combinations Where the Number of Instruments Is >2 eFigure 1. Forest Plots Showing the Single SNP Analysis for the Exposures of Gestational Hypertension and Pre-eclampsia or Eclampsia and the Outcome of Coronary Artery Disease eFigure 2. Forest Plots Showing the Single SNP Analysis for the Exposures of Hypertensive Disorders in Pregnancy, Gestational Hypertension and Pre-eclampsia or Eclampsia and the Outcome of Ischemic Stroke eFigure 3. Forest Plots Showing the Single SNP Analysis for the Exposures of Hypertensive Disorders in Pregnancy, Gestational Hypertension and Pre-eclampsia or Eclampsia and the Outcome of Heart Failure eFigure 4. Forest Plots Showing the Single SNP Analysis for the Exposures of Hypertensive Disorders in Pregnancy, Gestational Hypertension and Pre-eclampsia or Eclampsia and the Outcome of Atrial Fibrillation [file jamanetwopen-e230034-s001.pdf]

Supplementary Online Content

Rayes B, Ardissino M, Slob EAW, Patel KHK, Girling J, Ng FS. Association of hypertensive disorders of pregnancy with future cardiovascular disease. *JAMA Netw Open*. 2023;6(2):e230034. doi:10.1001/jamanetworkopen.2023.0034

eMethods. Detailed Methods

eTable 1. Information on the Studies and Consortia From Which Genetic Association Data Were Obtained

eTable 2. Assessment of Exposure-Mediator Associations

eTable 3. List of Instrumental Variables Used in the Analyses for the Exposure of Hypertensive Disorders of Pregnancy, and Corresponding Gene-Outcome Association Estimates for All SNPs Available in Outcome GWAS or Proxies Discovered Using a Linkage Disequilibrium Threshold of 0.8

eTable 4. List of Instrumental Variables Used in the Analyses for the Exposure of Gestational Hypertension, and Corresponding Gene-Outcome Association Estimates for All SNPs Available in Outcome GWAS or Proxies Discovered Using a Linkage Disequilibrium Threshold of 0.8

eTable 5. List of Instrumental Variables Used in the Analyses for the Exposure of Pre-eclampsia or Eclampsia, and Corresponding Gene-Outcome Association Estimates for All SNPs Available in Outcome GWAS or Proxies Discovered Using a Linkage Disequilibrium Threshold of 0.8

eTable 6. Results of Leave-1-Out Analyses for All Exposure-Outcome Combinations Where the Number of Instruments Is >2

eFigure 1. Forest Plots Showing the Single SNP Analysis for the Exposures of Gestational Hypertension and Pre-eclampsia or Eclampsia and the Outcome of Coronary Artery Disease

eFigure 2. Forest Plots Showing the Single SNP Analysis for the Exposures of Hypertensive Disorders in Pregnancy, Gestational Hypertension and Pre-eclampsia or Eclampsia and the Outcome of Ischemic Stroke

eFigure 3. Forest Plots Showing the Single SNP Analysis for the Exposures of Hypertensive Disorders in Pregnancy, Gestational Hypertension and Pre-eclampsia or Eclampsia and the Outcome of Heart Failure

eFigure 4. Forest Plots Showing the Single SNP Analysis for the Exposures of Hypertensive Disorders in Pregnancy, Gestational Hypertension and Pre-eclampsia or Eclampsia and the Outcome of Atrial Fibrillation

This supplementary material has been provided by the authors to give readers additional information about their work.

© 2023 Rayes B et al. *JAMA Network Open.*

eMethods. Detailed Methods

Assumptions

The key assumptions of Mendelian randomization analyses are as follows:
i) That the genetic variant is associated with the exposure
ii) That the variant is not associated with the outcome via a confounding pathway
iii) That the variant is associated with the outcome only through the exposure, and not through parallel biological pathways (horizontal pleiotropy)

Calculation of F-statistics and R^2 measures

R^2 represents the proportion of variability in the exposure that is explained by the genotype and was calculated for each SNP using the formula shown below:

$$\boldsymbol{R^2_{SNP}} = \frac{\boldsymbol{2\beta^2 MAF(1-MAF)}}{\boldsymbol{2\beta^2 MAF(1-MAF) + 2nMAF(1-MAF)se^2}}$$

β represents the effect size of the genetic variant in the exposure GWAS, $\boldsymbol{MAF}$ represents the allele frequency of the variant, $\boldsymbol{se}$ represents the standard error of the effect size of the genetic variant and $\boldsymbol{n}$ represents the sample size.
To obtain the total $\boldsymbol{R^2}$ for each exposure, we take the sum of $\boldsymbol{R^2_{SNP}}$ over all SNPs, since the SNPs are clumped and hence they can be treated as independent.

To assess the strength of the IVs, the F-statistic was calculated using the formula

$$\boldsymbol{F} = \frac{\boldsymbol{R^2/k}}{\boldsymbol{(1-R^2)/(n-k-1)}}$$

where $\boldsymbol{k}$ is the number of IVs and $\boldsymbol{R^2}$ is the summed SNP-wise $\boldsymbol{R^2}$.

Sensitivity Analyses

MR-Egger regression introduces an intercept term in the regression model which represents the average pleiotropic effect and accounts for directional pleiotropy. It provides an estimate of the causal effect under the InSIDE (Instrument Strength Independent of Direct Effect) assumption: the condition that the weighted correlation between the pleiotropic (direct) effect of the genetic variant on the outcome and the genetic association with the exposure is zero. The weighted median method takes the median of the ratio estimates rather than the weighted mean as with IVW MR. This can provide consistent estimates provided that over 50% of the genetic instruments are valid IVs. MR-PRESSO identifies outlier SNPs and provides effect estimates after adjustment for potential outliers.

© 2023 Rayes B et al. *JAMA Network Open.*

eTable 1: Information on the studies and consortia from which genetic association data were obtained. EUR = European, ICD = international classification of diseases, PMID = PubMed ID, UKB = UK Biobank, HUNT = Nord-Trøndelag Health Study

Phenotype	Study or consortium	Ancestry	Cases / controls	Case definition	Control definition	Units	Link / PMID
**Exposures**							
**Hypertensive disorder in pregnancy**	FinnGen	EUR	10,736 / 136,325	Cause of death or hospital discharge ICD-10: O10, O11, O13, O14, O15, O16; ICD-9: 642; ICD-8: 63701 63703 63704 63709 63710 63799	No past hypertensive disorder in pregnancy; parous	Log(OR)	https://www.finngen.fi/en/access_results
**Gestational hypertension**	FinnGen	EUR	5,240 / 136,235	Cause of death or hospital discharge ICD-10: O13; ICD-9: 6423; ICD-8: 63701	No past hypertensive disorder in pregnancy; parous	Log(OR)	
**Pre-eclampsia / eclampsia**	FinnGen	EUR	4,743 / 136,235	Cause of death or hospital discharge ICD-10: O11, O14, O15.0, O15.1, O15.2; ICD-9: 642[4-7]; ICD-8: 6370[349] 63710 63799 66120	No past hypertensive disorder in pregnancy; parous	Log(OR)	
**Outcomes**							
**Coronary artery disease**	Van der Harst et al	EUR	122,733 /424,528	Coronary artery disease or myocardial infarction	No known coronary artery disease or past myocardial infarction	Log(OR)	29212778
**Ischaemic stroke**	Malik et al	EUR	34,217 /406,111	Any ischaemic stroke	No history of stroke, of any type	Log(OR)	29531354
**Heart failure**	Shah et al	EUR	47,309 /930,014	Diagnosis of heart failure by physician, or healthcare record, and corroborated on self-report	No history of heart failure	Log(OR)	21378990
**Atrial fibrillation**	Nielsen et al	EUR	60,620 /970,216	Clinically diagnosed atrial fibrillation or flutter UKB and HUNT cohorts: ICD-9 427.3 ICD-10 I48	No history of atrial fibrillation, flutter or other arrhythmias	Log(OR)	30061737
**Mediators**							
**Body mass index**	Pulit et al	EUR	434,794	n/a	n/a	1-SD	30239722
**Type 2 diabetes**	Mahajan et al	EUR	80,154 /853,816	n/a	n/a	Log(OR)	35551307
**Systolic blood pressure**	Evangelou et al	EUR	757,601	n/a	n/a	1-mmHg	30224653

© 2023 Rayes B et al. *JAMA Network Open.*

eTable 2: Assessment of exposure-mediator associations

Exposure	Outcome	MR Method	SNP number	Beta	SE	P value
Any hypertensive disorder in pregnancy	BMI	IVW	4	-0.04	0.02	0.115
	Systolic BP	IVW	4	6.93	0.457	4.78×10^{-52}
	T2DM	IVW	3	0.08	0.05	0.072
Gestational hypertension	BMI	IVW	2	0.01	0.01	0.417
	Systolic BP	IVW	3	2.05	1.37	0.135
	T2DM	IVW	3	-0.01	0.03	0.770

BMI indicates body mass index; BP, blood pressure; IVW, inverse-variance weighted; MR, Mendelian randomization; SE, standard error; SNP, single nucleotide polymorphism; T2DM, type 2 diabetes mellitus

© 2023 Rayes B et al. *JAMA Network Open.*

eTable 3: List of instrumental variables used in the analyses for the exposure of hypertensive disorders of pregnancy, and corresponding gene-outcome association estimates for all SNPs available in outcome GWAS or proxies discovered using a linkage disequilibrium threshold of 0.8. SNP = single nucleotide polymorphism, eaf = effect allele frequency, se= standard error, pval = p value, ncase = number of cases, ncontrol = number of controls, rsq = R-Squared, fst = F-statistic, chr = chromosome, pos = position.

**Hypertensive disorders of pregnancy**												
**SNP**	**effect_allele**	**other_allele**	**eaf**	**beta**	**se**	**pval**	**ncase**	**ncontrol**	**rsq**	**fst**		
rs10857147	T	A	0.3130	0.1103	0.0162	1.20x10^{-11}	10736	136325	0.0043	46.0		
rs10882398	A	T	0.5940	0.0886	0.0153	6.81x10^{-9}	10736	136325	0.0031	33.6		
rs167479	G	T	0.5470	0.0923	0.0152	1.19x10^{-9}	10736	136325	0.0034	37.0		
rs17367504	G	A	0.1440	-0.1323	0.0214	6.77x10^{-10}	10736	136325	0.0035	38.1		
**Atrial fibrillation**												
**SNP**	**effect_allele**	**other_allele**	**palindromic**	**ambiguous**	**chr**	**pos**	**beta**	**se**	**pval**	**proxy**	**target_snp**	**proxy_snp**
rs10857147	T	A	TRUE	FALSE	4	81181072	0.0401	0.0074	5.60x10^{-8}	NA	NA	NA
rs10882398	A	T	TRUE	TRUE	10	95892788	-0.0010	0.0067	0.8878	NA	NA	NA
rs17367504	G	A	FALSE	FALSE	1	11862778	0.0232	0.0091	0.0109	NA	NA	NA
**Coronary artery disease**												
**SNP**	**effect_allele**	**other_allele**	**palindromic**	**ambiguous**	**chr**	**pos**	**beta**	**se**	**pval**	**proxy**	**target_snp**	**proxy_snp**
rs10857147	T	A	TRUE	TRUE	4	81181072	0.0463	0.0078	2.30x10^{-9}	NA	NA	NA
rs10882398	A	T	TRUE	TRUE	10	95892788	0.0026	0.0071	0.7200	NA	NA	NA
rs17367504	G	A	FALSE	FALSE	1	11862778	-0.0286	0.0094	0.0024	NA	NA	NA
**Heart failure**												
**SNP**	**effect_allele**	**other_allele**	**palindromic**	**ambiguous**	**chr**	**pos**	**beta**	**se**	**pval**	**proxy**	**target_snp**	**proxy_snp**
rs10857147	T	A	TRUE	TRUE	4	81181072	0.0288	0.0102	4.84x10^{-3}	NA	NA	NA
rs10882398	A	T	TRUE	TRUE	10	95892788	-0.0044	0.0080	0.5808	NA	NA	NA
rs167479	G	T	FALSE	FALSE	19	11526765	0.0103	0.0090	0.2562	NA	NA	NA
rs17367504	G	A	FALSE	FALSE	1	11862778	0.0178	0.0108	0.1001	NA	NA	NA
**Ischaemic stroke**												
**SNP**	**effect_allele**	**other_allele**	**palindromic**	**ambiguous**	**chr**	**pos**	**beta**	**se**	**pval**	**proxy**	**target_snp**	**proxy_snp**
rs10857147	T	A	TRUE	FALSE	4	81181072	0.0336	0.0105	1.32x10^{-3}	NA	NA	NA
rs10882398	A	T	TRUE	TRUE	10	95892788	0.0103	0.0088	0.2434	NA	NA	NA
rs17367504	G	A	FALSE	FALSE	1	11862778	-0.0234	0.0119	0.0504	NA	NA	NA

© 2023 Rayes B et al. *JAMA Network Open.*

eTable 4: List of instrumental variables used in the analyses for the exposure of gestational hypertension, and corresponding gene-outcome association estimates for all SNPs available in outcome GWAS or proxies discovered using a linkage disequilibrium threshold of 0.8. SNP = single nucleotide polymorphism, eaf = effect allele frequency, se= standard error, pval = p value, ncase = number of cases, ncontrol = number of controls, rsq = R-Squared, fst = F-statistic, chr = chromosome, pos = position.

**Gestational hypertension**													
**SNP**	**effect_allele**	**other_allele**	**eaf**	**beta**	**se**	**pval**	**ncase**	**ncontrol**	**rsq**	**fst**			
rs181872067	A	G	0.0103	0.5961	0.1093	$4.86x10^{-8}$	5240	136325	0.0057	29.8			
rs12656497	C	T	0.5860	0.1204	0.0211	$1.21x10^{-8}$	5240	136325	0.0061	32.5			
rs2208589	G	A	0.8880	0.1976	0.0340	$6.33x10^{-9}$	5240	136325	0.0064	33.7			
**Atrial fibrillation**													
**SNP**	**effect_allele**	**other_allele**	**palindromic**	**ambiguous**	**chr**	**pos**	**beta**	**se**	**pval**	**proxy**	**target_snp**	**proxy_snp**	
rs12656497	C	T	FALSE	FALSE	5	32831939	0.0213	0.0067	$1.63x10^{-3}$	NA	NA	NA	
rs181872067	A	G	FALSE	FALSE	2	8825572	-0.0042	0.0311	0.8934	NA	NA	NA	
rs2208589	G	A	FALSE	FALSE	20	47408414	0.0158	0.0083	0.0568	NA	NA	NA	
**Coronary artery disease**													
**SNP**	**effect_allele**	**other_allele**	**palindromic**	**ambiguous**	**chr**	**pos**	**beta**	**se**	**pval**	**proxy**	**target_snp**	**proxy_snp**	
rs12656497	C	T	FALSE	FALSE	5	32831939	0.0156	0.0071	0.0270	NA	NA	NA	
rs181872067	A	G	FALSE	FALSE	2	8825572	0.0015	0.0293	0.9600	NA	NA	NA	
rs2208589	G	A	FALSE	FALSE	20	47408414	0.0222	0.0085	0.0087	NA	NA	NA	
**Heart failure**													
**SNP**	**effect_allele**	**other_allele**	**palindromic**	**ambiguous**	**chr**	**pos**	**beta**	**se**	**pval**	**proxy**	**target_snp**	**proxy_snp**	
rs12656497	C	T	FALSE	FALSE	5	32831939	0.0098	0.0080	0.2165	NA	NA	NA	
rs181872067	A	G	FALSE	FALSE	2	8825572	-0.0084	0.0357	0.8143	NA	NA	NA	
rs2208589	G	A	FALSE	FALSE	20	47408414	0.0131	0.0098	0.1818	NA	NA	NA	
**Ischaemic stroke**													
**SNP**	**effect_allele**	**other_allele**	**palindromic**	**ambiguous**	**chr**	**pos**	**beta**	**se**	**pval**	**proxy**	**target_snp**	**proxy_snp**	
rs12656497	C	T	FALSE	FALSE	5	32831939	0.0302	0.0086	$4.60x10^{-4}$	NA	NA	NA	
rs2208589	G	A	FALSE	FALSE	20	47408414	0.0090	0.0102	0.3783	NA	NA	NA	

© 2023 Rayes B et al. *JAMA Network Open.*

eTable 5: List of instrumental variables used in the analyses for the exposure of pre-eclampsia or eclampsia, and corresponding gene-outcome association estimates for all SNPs available in outcome GWAS or proxies discovered using a linkage disequilibrium threshold of 0.8. SNP = single nucleotide polymorphism, eaf = effect allele frequency, se= standard error, pval = p value, ncase = number of cases, ncontrol = number of controls, rsq = R-Squared, fst = F-statistic, chr = chromosome, pos = position.

**Pre-eclampsia or eclampsia**												
**SNP**	**effect_allele**	**other_allele**	**eaf**	**beta**	**se**	**pval**	**ncase**	**ncontrol**	**rsq**	**fst**		
rs10004588	A	C	0.0452	0.2421	0.0525	3.97x10^{-6}	4743	136325	0.0045	21.4		
rs10944316	T	C	0.0695	0.2003	0.0430	3.17x10^{-6}	4743	136325	0.0046	21.7		
rs11121976	T	C	0.1620	-0.1421	0.0292	1.13x10^{-6}	4743	136325	0.0050	23.7		
rs113653429	C	T	0.0336	0.2720	0.0595	4.86x10^{-6}	4743	136325	0.0044	20.9		
rs116887748	T	C	0.0375	0.2594	0.0567	4.85x10^{-6}	4743	136325	0.0044	20.9		
rs1226832	C	G	0.1270	-0.1545	0.0325	2.04x10^{-6}	4743	136325	0.0047	22.6		
rs12775642	A	G	0.3230	0.1135	0.0231	9.22x10^{-7}	4743	136325	0.0051	24.1		
rs137882343	T	G	0.0112	0.5126	0.1062	1.38x10^{-6}	4743	136325	0.0049	23.3		
rs138609024	C	T	0.0063	0.7384	0.1501	8.67x10^{-7}	4743	136325	0.0051	24.2		
rs167479	G	T	0.5740	0.1082	0.0217	6.38x10^{-7}	4743	136325	0.0052	24.8		
rs17367504	G	A	0.1450	-0.1498	0.0307	1.03x10^{-6}	4743	136325	0.0050	23.9		
rs17572606	T	C	0.0099	0.5406	0.1147	2.42x10^{-6}	4743	136325	0.0047	22.8		
rs2369286	A	G	0.2550	-0.1144	0.0247	3.75x10^{-6}	4743	136325	0.0045	21.4		
rs2912370	C	T	0.5210	-0.0999	0.0217	4.19x10^{-6}	4743	136325	0.0044	21.2		
rs4766568	C	T	0.1600	-0.1411	0.0297	2.00x10^{-6}	4743	136325	0.0047	22.6		
rs6060809	T	C	0.0348	0.2698	0.0588	4.47x10^{-6}	4743	136325	0.0044	21.1		
rs60736424	C	T	0.2840	-0.1104	0.0239	4.01x10^{-6}	4743	136325	0.0045	21.3		
rs7388321	C	G	0.9710	-0.3141	0.0641	9.67x10^{-7}	4743	136325	0.0050	24.0		
**Atrial fibrillation**												
**SNP**	**effect_allele**	**other_allele**	**palindromic**	**ambiguous**	**chr**	**pos**	**beta**	**se**	**pval**	**proxy**	**target_snp**	**proxy_snp**
rs10004588	A	C	FALSE	FALSE	4	126390454	0.0028	0.0138	0.8369	NA	NA	NA
rs10944316	T	C	FALSE	FALSE	6	88261817	0.0690	0.0188	2.52x10^{-4}	NA	NA	NA
rs113653429	C	T	FALSE	FALSE	1	21557407	-0.0060	0.0210	0.7744	NA	NA	NA
rs116887748	T	C	FALSE	FALSE	18	60358234	-0.0234	0.0292	0.4240	NA	NA	NA
rs1226832	C	G	TRUE	FALSE	1	45595626	-0.0151	0.0104	0.1456	NA	NA	NA
rs12775642	A	G	FALSE	FALSE	10	121712667	-0.0281	0.0073	1.34x10^{-4}	NA	NA	NA
rs137882343	T	G	FALSE	FALSE	18	65010976	0.0356	0.0259	0.1687	NA	NA	NA
rs138609024	C	T	FALSE	FALSE	17	32119136	0.0958	0.0345	5.50x10^{-3}	NA	NA	NA
rs17367504	G	A	FALSE	FALSE	1	11862778	0.0232	0.0091	0.0109	NA	NA	NA

© 2023 Rayes B et al. *JAMA Network Open.*

rs17572606	T	C	FALSE	FALSE	22	24868172	-0.0146	0.0303	0.6312	NA	NA	NA
rs2369286	A	G	FALSE	FALSE	4	4599145	$5.00x10^{-4}$	0.0077	0.9501	TRUE	rs2369286	rs28858968
rs2912370	C	T	FALSE	FALSE	15	39050654	0.0086	0.0070	0.2155	NA	NA	NA
rs4766568	C	T	FALSE	FALSE	12	111724699	-0.0042	0.0093	0.6526	NA	NA	NA
rs6060809	T	C	FALSE	FALSE	20	34717350	0.0164	0.0184	0.3738	NA	NA	NA
rs60736424	C	T	FALSE	FALSE	6	154936452	0.0023	0.0126	0.8525	NA	NA	NA
rs7388321	C	G	TRUE	FALSE	8	17939143	-0.0237	0.0520	0.6478	NA	NA	NA
**Coronary artery disease**												
**SNP**	**effect_allele**	**other_allele**	**palindromic**	**ambiguous**	**chr**	**pos**	**beta**	**se**	**pval**	**proxy**	**target_snp**	**proxy_snp**
rs10004588	A	C	FALSE	FALSE	4	126390454	0.0418	0.0149	0.0050	NA	NA	NA
rs10944316	T	C	FALSE	FALSE	6	88261817	0.0261	0.0194	0.1800	NA	NA	NA
rs113653429	C	T	FALSE	FALSE	1	21557407	-0.0112	0.0216	0.6100	NA	NA	NA
rs116887748	T	C	FALSE	FALSE	18	60358234	0.0560	0.0321	0.0810	NA	NA	NA
rs1226832	C	G	TRUE	TRUE	1	45595626	0.0076	0.0106	0.4700	NA	NA	NA
rs12775642	A	G	FALSE	FALSE	10	121712667	0.0019	0.0075	0.8100	NA	NA	NA
rs137882343	T	G	FALSE	FALSE	18	65010976	0.0201	0.0245	0.4100	NA	NA	NA
rs138609024	C	T	FALSE	FALSE	17	32119136	-0.0203	0.0297	0.4900	NA	NA	NA
rs17367504	G	A	FALSE	FALSE	1	11862778	-0.0286	0.0094	0.0024	NA	NA	NA
rs2369286	A	G	FALSE	FALSE	4	4595540	0.0047	0.0078	0.5500	NA	NA	NA
rs2912370	C	T	FALSE	FALSE	15	39050654	0.0066	0.0071	0.3500	NA	NA	NA
rs4766568	C	T	FALSE	FALSE	12	111724699	-0.0317	0.0099	0.0014	NA	NA	NA
rs6060809	T	C	FALSE	FALSE	20	34717350	0.0400	0.0181	0.0280	NA	NA	NA
rs60736424	C	T	FALSE	FALSE	6	154937373	-0.0130	0.0082	0.1200	TRUE	rs60736424	rs10782335
rs7388321	C	G	TRUE	TRUE	8	17939143	-0.0212	0.0441	0.6300	NA	NA	NA
**Heart failure**												
**SNP**	**effect_allele**	**other_allele**	**palindromic**	**ambiguous**	**chr**	**pos**	**beta**	**se**	**pval**	**proxy**	**target_snp**	**proxy_snp**
rs10004588	A	C	FALSE	FALSE	4	126390454	0.0033	0.0157	0.8314	NA	NA	NA
rs10944316	T	C	FALSE	FALSE	6	88261817	0.0355	0.0212	0.0947	NA	NA	NA
rs11121976	T	C	FALSE	FALSE	1	12833428	-0.0059	0.0103	0.5662	NA	NA	NA
rs113653429	C	T	FALSE	FALSE	1	21557407	0.0297	0.0230	0.1959	NA	NA	NA
rs116887748	T	C	FALSE	FALSE	18	60358234	0.0479	0.0321	0.1356	NA	NA	NA
rs1226832	C	G	TRUE	TRUE	1	45595626	-0.0102	0.0120	0.3968	NA	NA	NA
rs12775642	A	G	FALSE	FALSE	10	121712667	-0.0167	0.0087	0.0545	NA	NA	NA
rs137882343	T	G	FALSE	FALSE	18	65010976	0.0202	0.0300	0.4998	NA	NA	NA
rs167479	G	T	FALSE	FALSE	19	11526765	0.0103	0.0090	0.2562	NA	NA	NA

© 2023 Rayes B et al. *JAMA Network Open.*

rs17367504	G	A	FALSE	FALSE	1	11862778	0.0178	0.0108	0.1001	NA	NA	NA
rs17572606	T	C	FALSE	FALSE	22	24868172	-0.0094	0.0353	0.7893	NA	NA	NA
rs2369286	A	G	FALSE	FALSE	4	4595540	-0.0208	0.0088	0.0182	NA	NA	NA
rs2912370	C	T	FALSE	FALSE	15	39050654	-0.0061	0.0081	0.4542	NA	NA	NA
rs4766568	C	T	FALSE	FALSE	12	111724699	-0.0199	0.0111	0.0726	NA	NA	NA
rs6060809	T	C	FALSE	FALSE	20	34717350	0.0368	0.0211	0.0809	NA	NA	NA
rs60736424	C	T	FALSE	FALSE	6	154936452	0.0079	0.0132	0.5521	NA	NA	NA
rs7460992	T	C	FALSE	FALSE	8	17929475	-0.0168	0.0297	0.5712	NA	NA	NA
**Ischaemic stroke**												
**SNP**	**effect_allele**	**other_allele**	**palindromic**	**ambiguous**	**chr**	**pos**	**beta**	**se**	**pval**	**proxy**	**target_snp**	**proxy_snp**
rs10004588	A	C	FALSE	FALSE	4	126390454	0.0141	0.0176	0.4213	NA	NA	NA
rs10944316	T	C	FALSE	FALSE	6	88261817	0.0526	0.0275	0.0555	NA	NA	NA
rs113653429	C	T	FALSE	FALSE	1	21557407	-0.0441	0.0286	0.1233	NA	NA	NA
rs1226832	C	G	TRUE	FALSE	1	45595626	-0.0039	0.0149	0.7917	NA	NA	NA
rs12775642	A	G	FALSE	FALSE	10	121712667	-0.0099	0.0097	0.3082	NA	NA	NA
rs17367504	G	A	FALSE	FALSE	1	11862778	-0.0234	0.0119	0.0504	NA	NA	NA
rs2369286	A	G	FALSE	FALSE	4	4595540	-0.0027	0.0095	0.7749	NA	NA	NA
rs2912370	C	T	FALSE	FALSE	15	39050654	-0.0035	0.0093	0.7055	NA	NA	NA
rs4766568	C	T	FALSE	FALSE	12	111724699	-0.0423	0.0109	0.0001	NA	NA	NA
rs6060809	T	C	FALSE	FALSE	20	34717350	-0.0019	0.0240	0.9382	NA	NA	NA
rs60736424	C	T	FALSE	FALSE	6	154936452	0.0015	0.0105	0.8877	NA	NA	NA
rs7460992	T	C	FALSE	FALSE	8	17929475	0.0044	0.0146	0.7653	NA	NA	NA

© 2023 Rayes B et al. *JAMA Network Open.*

eTable 6: Results of leave-1-out analyses for all exposure-outcome combinations where the number of instruments is > 2. SNP = single nucleotide polymorphism, b = beta, se = standard error, pval = p value

Outcome	SNP removed	b	se	pval
**Atrial fibrillation**	**Gestational hypertension**			
	rs12656497	0.046	0.042	0.282
	rs181872067	0.115	0.047	0.014
	rs2208589	0.079	0.092	0.390
	**Pre-eclampsia or eclampsia**			
	rs10004588	0.009	0.033	0.790
	rs10944316	-0.002	0.028	0.942
	rs113653429	0.011	0.032	0.743
	rs116887748	0.011	0.032	0.722
	rs1226832	0.003	0.032	0.921
	rs12775642	0.028	0.027	0.296
	rs137882343	0.002	0.033	0.962
	rs138609024	-0.009	0.031	0.778
	rs17367504	0.023	0.030	0.455
	rs17572606	0.013	0.033	0.703
	rs2369286	0.010	0.033	0.761
	rs2912370	0.015	0.032	0.643
	rs4766568	0.008	0.033	0.816
	rs6060809	0.006	0.032	0.860
	rs60736424	0.010	0.032	0.761
	rs7388321	0.008	0.032	0.792
**Coronary artery disease**	**Gestational hypertension**			
	rs12656497	0.065	0.055	0.233
	rs181872067	0.119	0.035	0.001
	rs2208589	0.055	0.063	0.381
	**Pre-eclampsia or eclampsia**			
	rs10004588	0.052	0.029	0.069
	rs10944316	0.060	0.030	0.043
	rs113653429	0.068	0.029	0.020
	rs116887748	0.059	0.029	0.040
	rs12775642	0.066	0.030	0.029
	rs137882343	0.066	0.032	0.036
	rs138609024	0.085	0.029	0.004
	rs17367504	0.051	0.028	0.069
	rs2369286	0.070	0.029	0.016
	rs2912370	0.071	0.029	0.013
	rs4766568	0.051	0.027	0.060
	rs6060809	0.056	0.030	0.058
	rs60736424	0.059	0.030	0.048
**Heart failure**	**Gestational hypertension**			
	rs12656497	0.034	0.039	0.395

© 2023 Rayes B et al. *JAMA Network Open.*

	rs181872067	0.072	0.040	0.071
	rs2208589	0.029	0.047	0.546
	**Pre-eclampsia or eclampsia**			
	rs10004588	0.047	0.027	0.077
	rs10944316	0.039	0.025	0.117
	rs11121976	0.044	0.027	0.095
	rs113653429	0.040	0.026	0.119
	rs116887748	0.040	0.025	0.108
	rs12775642	0.058	0.022	0.010
	rs137882343	0.045	0.027	0.099
	rs167479	0.041	0.026	0.115
	rs17367504	0.057	0.023	0.014
	rs17572606	0.050	0.026	0.055
	rs2369286	0.034	0.024	0.159
	rs2912370	0.043	0.026	0.102
	rs4766568	0.038	0.025	0.140
	rs6060809	0.038	0.026	0.139
	rs60736424	0.047	0.025	0.062
	rs7460992	0.044	0.026	0.094
**Ischemic stroke**	**Pre-eclampsia or eclampsia**			
	rs10004588	0.041	0.039	0.294
	rs10944316	0.036	0.035	0.308
	rs113653429	0.054	0.034	0.119
	rs1226832	0.044	0.038	0.248
	rs12775642	0.054	0.036	0.139
	rs17367504	0.031	0.037	0.391
	rs2369286	0.044	0.038	0.247
	rs2912370	0.043	0.038	0.256
	rs4766568	0.016	0.027	0.560
	rs6060809	0.046	0.038	0.220
	rs60736424	0.046	0.038	0.218
	rs7460992	0.058	0.040	0.146

© 2023 Rayes B et al. *JAMA Network Open.*

eFigure 1: Forest plots showing the single SNP analysis for the exposures of gestational hypertension and pre-eclampsia or eclampsia and the outcome of coronary artery disease. Single SNP analysis for hypertensive disorders of pregnancy is displayed in the primary analyses, since only one instrumental SNP was available after harmonisation.

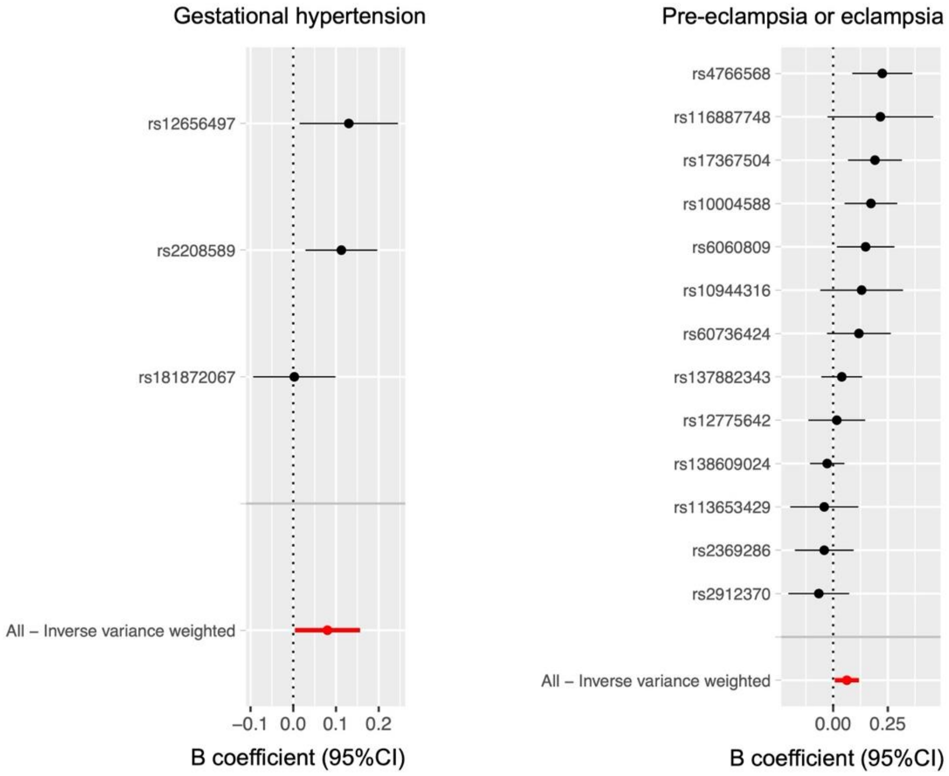

© 2023 Rayes B et al. *JAMA Network Open.*

eFigure 2: Forest plots showing the single SNP analysis for the exposures of hypertensive disorders in pregnancy, gestational hypertension and pre-eclampsia or eclampsia and the outcome of ischemic stroke

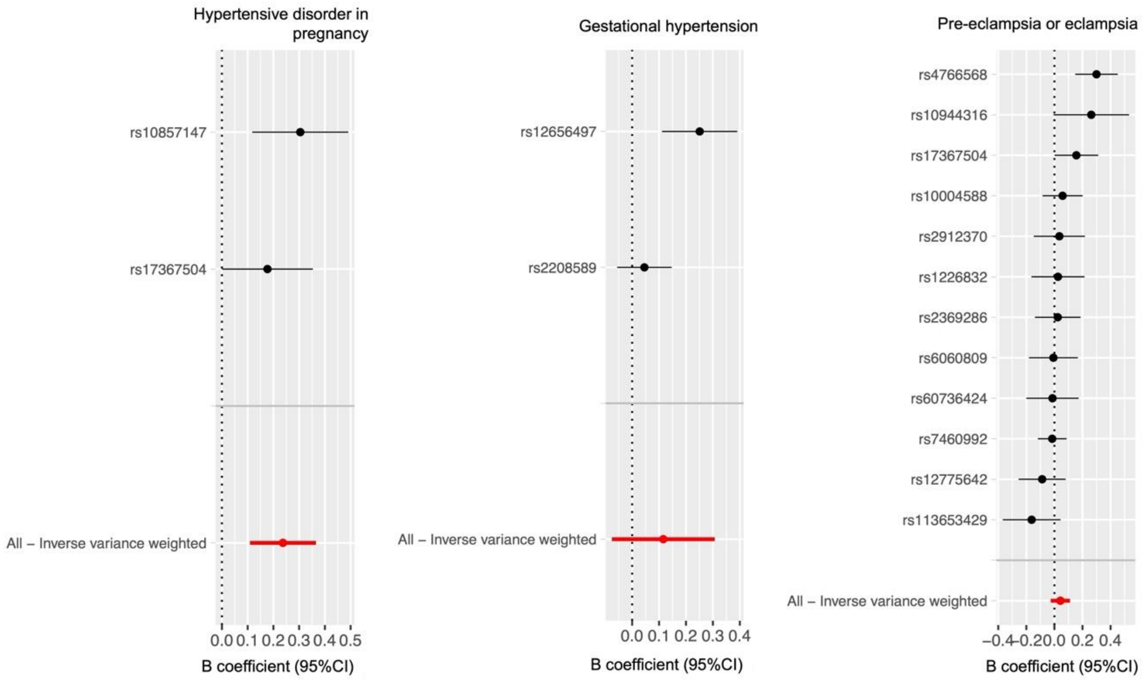

© 2023 Rayes B et al. *JAMA Network Open.*

eFigure 3: Forest plots showing the single SNP analysis for the exposures of hypertensive disorders in pregnancy, gestational hypertension and pre-eclampsia or eclampsia and the outcome of heart failure

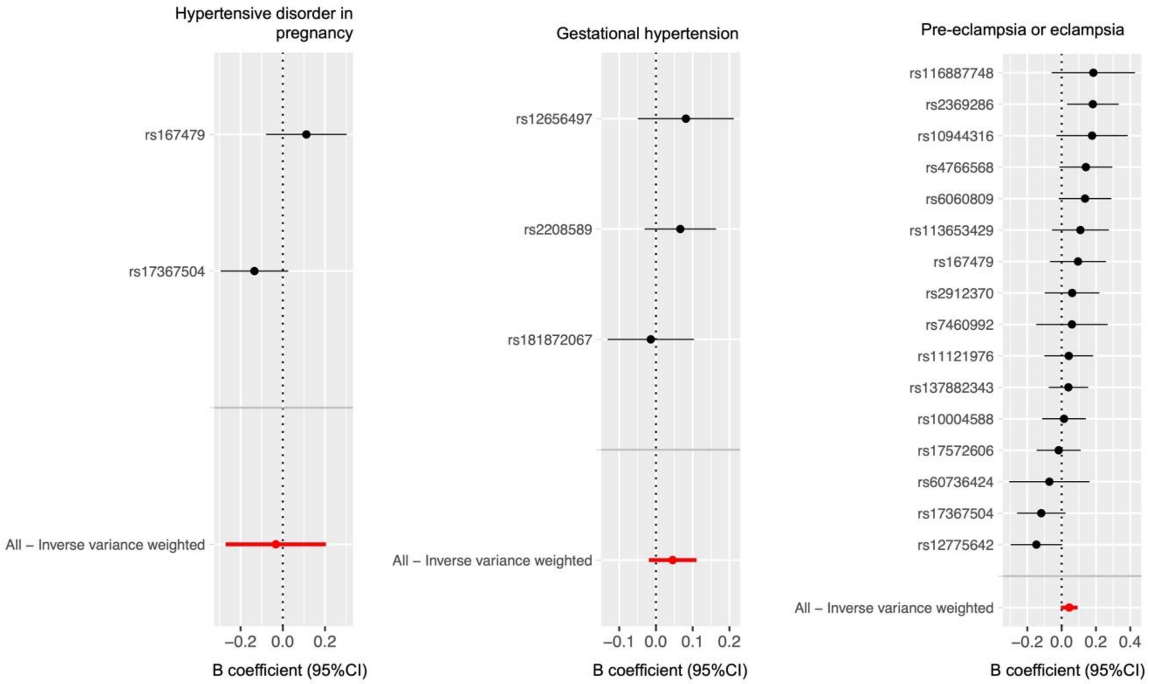

© 2023 Rayes B et al. *JAMA Network Open.*

e Figure 4: Forest plots showing the single SNP analysis for the exposures of hypertensive disorders in pregnancy, gestational hypertension and pre-eclampsia or eclampsia and the outcome of atrial fibrillation

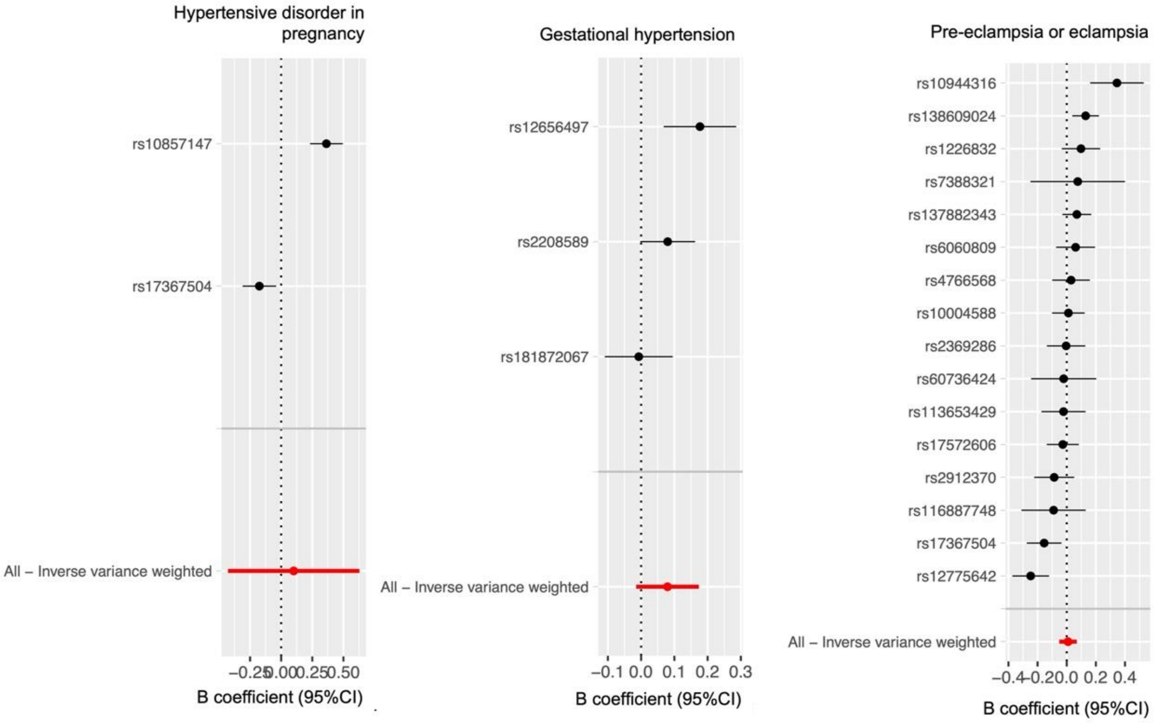

© 2023 Rayes B et al. *JAMA Network Open.*
