# Supplementary material for: Association of Hypertensive Disorders of Pregnancy With Future Cardiovascular Disease
Source: JAMA Netw Open. 2023 Feb 17;6(2):e230034. doi: 10.1001/jamanetworkopen.2023.0034 (PMC9938428; doi:10.1001/jamanetworkopen.2023.0034)
Supplement: Supplement 2. — Data Sharing Statement [file jamanetwopen-e230034-s002.pdf]

Data Sharing Statement

Rayes. Association of Hypertensive Disorders of Pregnancy With Future Cardiovascular Disease. *JAMA Netw Open*. Published February 17, 2023.
doi:10.1001/jamanetworkopen.2023.0034

Data
Data available: No

Additional Information
Explanation for why data not available: Data used in the study is publicly available at reported resources.
